# The relationship of air pollution and surrogate markers of endothelial dysfunction in a population-based sample of children

**DOI:** 10.1186/1471-2458-11-115

**Published:** 2011-02-18

**Authors:** Parinaz Poursafa, Roya Kelishadi, Ahmadreza Lahijanzadeh, Mohammadreza Modaresi, Shaghayegh Haghjouy Javanmard, Raheleh Assari, Mohammad Mehdi Amin, Faramarz Moattar, Abbasgholi Amini, Babak Sadeghian

**Affiliations:** 1 Faculty of Environment and Energy, Science and Research Branch, Islamic Azad University, Tehran, Iran; 2 Pediatrics Department, Child Health Promotion Research Center, Isfahan University of Medical Sciences, Isfahan, Iran; 3 Environmental Protection Department, Isfahan, Iran; 4 Pediatrics Department, Pediatric Prevention Research Center, Isfahan University of Medical Sciences, Isfahan, Iran; 5 Department of Physiology, Applied Physiology Research Center, Isfahan University of Medical Sciences, Isfahan, Iran; 6 Pediatrics Department, School of Medicine, Pediatric Prevention Research Center, Isfahan University of Medical Sciences, Isfahan, Iran; 7 Department of Environmental Health Engineering, Environment Research Center, Isfahan University of Medical Sciences, Isfahan, Iran

## Abstract

**Background:**

This study aimed to assess the relationship of air pollution and plasma surrogate markers of endothelial dysfunction in the pediatric age group.

**Methods:**

This cross-sectional study was conducted in 2009-2010 among 125 participants aged 10-18 years. They were randomly selected from different areas of Isfahan city, the second large and air-polluted city in Iran. The association of air pollutants' levels with serum thrombomodulin (TM) and tissue factor (TF) was determined after adjustment for age, gender, anthropometric measures, dietary and physical activity habits.

**Results:**

Data of 118 participants was complete and was analyzed. The mean age was 12.79 (2.35) years. The mean pollution standards index (PSI) value was at moderate level, the mean particular matter measuring up to 10 μm (PM_10_) was more than twice the normal level. Multiple linear regression analysis showed that TF had significant relationship with all air pollutants except than carbon monoxide, and TM had significant inverse relationship with ozone. The odds ratio of elevated TF was significantly higher in the upper vs. the lowest quartiles of PM_10_, ozone and PSI. The corresponding figures were in opposite direction for TM.

**Conclusions:**

The relationship of air pollutants with endothelial dysfunction and pro-coagulant state can be an important factor in the development of atherosclerosis from early life. This finding should be confirmed in future longitudinal studies. Concerns about the harmful effects of air pollution on children's health should be considered a top priority for public health policy; it should be underscored in primordial and primary prevention of chronic diseases.

## Background

Today, air pollution is one of the major health threats both in developing and developed countries [1]. Children are more sensitive than adults to health effects of air pollutants; this might be because of the developmental changes in their respiratory system and their higher number of breaths per minute than adults. Moreover, compared to adults they may spend more time outdoors, and their activity level is higher [2,3].

The harmful effects of air pollution on cardiovascular system are well-documented, but the underlying mechanisms remain to be determined. A recent experimental study showed for the first time that pulmonary exposure to the particulate matter (PM) within diesel exhaust enhances atherogenesis [4]. The human blood vessel endothelium is a sensitive target for air pollutants [5]. The interactions of the inflammation and coagulation systems are of the main mechanisms involved in impairment of endothelial function and eventually cardiovascular diseases [6].

The effect of air pollution on inflammation, oxidative stress and cardiovascular risk factors has been demonstrated not only in older adults [7,8], but also in young adults [9] as well as in children and adolescents [10,11].

The inflammation process stimulates the coagulation system, and result in increased secretion of tissue factor (TF). Endothelial function has key roles in anticoagulant and fibrinolytic systems. In vitro studies have demonstrated significant decrease in endogenous anticoagulation activity, thrombomodulin (TM), endothelial protein C receptor antigen and culture of endothelial cells during the inflammation process [12-14].

A growing body of evidence suggests that the effects of air pollution on the inflammation and the coagulation systems may have a role in endothelial dysfunction and in turn in the progression of cardiovascular diseases [15-17]. Findings of experimental studies suggest that exposure to air pollution may result in increase in TF and decrease in TM [18,19].

Atherogenesis starts from the fetal life through interrelations of traditional risk factors with inflammatory, immune, and endothelial biomarkers [20]. Air pollution has various harmful effects on this process from early life [21-23]. Studying the effects of environmental factors on early stages of atherosclerosis in early life can help identify the underlying mechanisms.

To the best of our knowledge, no previous human study had determined the association of air pollutants with TF and TM in the pediatric age group. In the current study, we aimed to determine the relationship of air pollution with TS and TM, as surrogate markers of endothelial dysfunction, in a population-based sample of children and adolescents.

## Methods

This cross-sectional study was conducted from November 2009 to February 2010 in Isfahan, which is the second large and air-polluted city in Iran. The study was approved in the Research Council &Ethics Committee of the School of Medicine, Isfahan University of Medical Sciences, Isfahan, Iran. It was conducted according to the Declaration of Helsinki. After providing detailed oral information, we obtained written informed consent from the parents and oral assent from participants.

### Participants

Those children and adolescents were eligible who were aged 10 to 18 years, lived for at least 6 months in areas of the city which had air pollution measurement stations, and their homes and schools were located less than 1 kilometer far from these stations. Those individuals who had a history of active or passive smoking, chronic disease, long-term medication use, or a history of acute infectious diseases in the past two weeks were not included in the study.

By considering a power of 95% and the statistical significance of 5%, the sample size was calculated as 110, but because of possible attrition of participants, the study was conducted on 125 students. To avoid socioeconomic bias, they were selected by multistage-random cluster sampling, with consideration to the proportion of the different types of schools (public/private).

### Study area

Isfahan is an industrial city with a population of near 1894382, located in the center of Iranian plateau, with an average altitude of 1500 m from the sea level bounded by NW-SE mountain range of 3000 m. The average monthly temperature is 16°C with maximum 29°C in July and minimum 3°C in December with mild winds from west and south. Also the air of the city of Isfahan is predominantly affected by industrial emissions and motor traffic which can lead to a buildup of elevated concentrations during stagnant conditions [24,25].

### Clinical study and laboratory methods

After inviting the selected students to a health center, a trained nurse completed a questionnaire on demographic data, and physical examination was done by the same trained general practitioner and under the supervision of the same pediatrician. Subcutaneous fat of the biceps and triceps muscles were measured with a skinfold caliper (Mojtahedi, Iran), the percent body fat was determined by bio-electrical impedence using a Body Fat Monitor (Omron HBF-300, Japan).

For assessment of dietary habits, the Healthy Eating Index (HEI) was computed as described before [10]. Physical activity level was assessed by the international Physical Activity Questionnaire for Children [26], previously validated in Iranian children [27].

While one of parents accompanied his or her child, blood samples were taken from the ante-cubital vein. Serums were obtained after centrifuging blood samples, and were kept frozen at -70°C until assayed and analyzed in the laboratory of the Applied Physiology Research Center affiliated to Isfahan University of Medical Sciences. Enzyme-linked immunosorbent assay (ELISA) kits from Abcam Company (UK) with code Ab46508 were used for measurement of TM, and TF ELISA kits R&D Company (UK) with code DCF300 for laboratory examinations.

### Air pollution data

The mean daily temperature, sunlight duration, humidity and wind speed were recorded. Data from 5 air pollution measurement stations in Isfahan city were recorded daily for the 7 days prior to blood sampling. Daily data pertaining to main air pollutants, i.e. sulfur dioxide (SO_2_), Ozone (O_3_), PM_10_, Nitrogen dioxide (NO_2_) and carbon monoxide (CO) as well as the Pollutant Standards Index (PSI) were recorded. The mean values of seven 24-hour means of air pollutants and PSI were considered for statistical analysis.

### Statistical analysis

SPSS for Windows (version 15:0, SPSS Inc., Chicago, IL) was used for data analysis. Analyses were initially stratified by gender, but as the differences were not significant, results are presented for girls and boys combined. We used log-transformed concentrations of variables to achieve normal distributions. The relationships of air pollutants with serum TM and TF were determined by Pearson correlation test. The associations between air pollutants and markers of endothelial dysfunction were assessed by multiple linear regression after adjustment for age, gender, body mass index, waist circumference, healthy eating index and physical activity level. The concentrations of biomarkers and air pollutants were categorized to quartiles, and the upper quartile was considered as elevated value. As there is no cutoff value to determine the high and low levels of TM and TF in the pediatric age group, we considered their lowest quartiles as low, and their highest quartiles as high levels. Then, we examined the association of the dichotomized concentrations of these biomarkers (upper quartile vs. lower quartile) across the quartiles of air pollutants by using logistic regression analysis after adjustment for the abovementioned confounders. The significance level was set at p < 0.05.

## Results

Data of 118 out of the125 students studied was complete and included in the statistical analysis. The study participants consisted of 57 (48.3%) boys and 61 (51.7%) girls with a mean age of 12.79 ± 2.35 years.

The mean (SD) of anthropometric and biochemical variables of the study participants is presented in Table 1. TF had a mean level of 64.77 ± 17.35 pg/mL, with the following quartile (Q) ranges: Q1: 27.11-63.79; Q2:63.80-78.84; Q3:78.85-89.17; Q4: 89.18-102.74. The corresponding figures for TM were 5.64 ± 3.27 ng/mL, Q1: 2.18-5.14; Q2: 5.15-9.27; Q3: 9.28-14.25; Q4:14.26-15.71, respectively.

**Table 1:** Characteristics of study participants

Variables	Mean (SD)
**Age (years)**	12.79 (2.35)
**Body mass index (kg/m**^**2 **^**)**	20.64 (3.36)
**Waist circumference (cm)**	72.61 (4.75)
**Percent body fat (%)**	22.84 (4.27)
**Fat mass (kg)**	12.45 (2.56)
**Subcutaneous fat (mm)**	10.91 (2.17)
**Tissue Factor (pg/mL)**	0.71(0.11)
**Thrombomodulin (μmol/L)**	5.64(3.27)

SD: standard deviation

The environmental characteristics are presented in Table 2. It shows moderate levels of mean PSI, i.e. an inappropriate level for sensitive groups. Mean levels of O_3_, NO_2 _and SO_2 _were higher than acceptable values; the mean PM_10 _level was remarkably high, reaching more than twice the normal level (120.48 vs. 50 μg/m^3^).

**Table 2:** Environmental characteristics during the study period

	Mean(SD)	Range
**Temperature **(°C)	9.35 (4.61)	-1.1-15.4
**Humidity **(%)	49.45(5.17)	22.10-76.15
**Wind speed **(Km/h)	10.25 (2.65)	5.41-21.25
**Sunlight duration **(h)	8.45 (1.15)	7.05-11.10
**PM**_**10 **_(μg/m^3^)	120.48 (62.83)	44.58-284.12
**CO **(ppm)	3.91(2.51)	1.43-10.90
**SO**_**2 **_(ppb)	43.72(30.56)	5.50-89.9
**NO**_**2 **_(ppb)	59.39(35.55)	20.60-121.85
**O**_**3**_(ppb)	33.60 (10.23)	11.18-56.81
**PSI**	74.61(30.35)	39-136.2

SD: standard deviation; PM_10_: particular matter 10 (acceptable level: 50 μg/m^3^); CO: carbon monoxide (acceptable level: 9 ppm); SO_2_: sulfur dioxide (acceptable level: 0.03 ppb)

NO_2_: Nitrogen dioxide (acceptable level: 0.05 ppb); O_3: _ozone (acceptable level: 0.08 ppb)

PSI; Pollution Standards Index (0-50: Good; 51-100: Moderate; 101-199: Unhealthful; 200-299: Very unhealthful; ≥ 300: Hazardous)

Results of the Pearson correlation analyses of air pollutants level with serum markers showed that PSI and PM_10 _had significant correlation with TF (r = 0.3, p = 0.001) and non-significant inverse correlation with TM. CO had weak but significant correlation with TM and TF (r = 0.25, p = 0.01).

Multiple linear regression analysis showed that after adjustment for confounding factors, serum TF level had significant relationship with PSI. This relationship existed more or less with air pollutants, notably PM_10 _(Table 3).

**Table 3:** Regression coefficients* for the relation of air pollutants and Pollutant Standards Index with serum concentrations of biomarkers

	Tissue factor				Thrombomodulin			
	**Unstandardized Coefficients**		**Standardized Coefficients**	**P value**	**Unstandardized Coefficients**		**Standardized Coefficients**	**Pvalue**
	**Beta**	**SE**	**Beta**		**Beta**	**SE**	**Beta**	
**PSI**	0.45	0.07	0.55	<0.0001	-0.12	0.01	-0.16	0.15
**O**_**3**_	0.39	0.16	0.48	0.04	-0.38	0.03	-0.47	0.01
**NO**_**2**_	0.31	0.04	0.37	0.02	-0.21	0.01	-0.25	0.08
**PM**_**10**_	0.40	0.03	0.51	0.001	-0.34	0.08	-0.35	0.06
**SO**_**2**_	0.32	0.06	0.41	0.04	0.10	0.01	0.17	0.65
**CO**	0.37	0.07	0.39	0.52	-0.28	0.15	-0.21	0.07

*: Adjusted for age, gender, anthropometric measures, as well as dietary and physical activity habits

SE: standard error; PM_10_: particular matter 10 ((μg/m^3^); CO: carbon monoxide(ppm); SO_2_: sulfur dioxide (ppb); NO_2_: Nitrogen dioxide (ppb); O_3: _ozone(ppb); PSI; Pollution Standards Index

The odds ratio (OR) of elevated TF increased as the quartiles of PM_10_, O_3 _and PSI increased; however these associations reached to significant level only in the highest quartile of PM_10 _and PSI. The corresponding figures for TM were in opposite direction, i.e. the OR was lower in the highest vs. lowest quartiles of PM10, O_3 _and PSI (Table 4).

**Table 4:** Associatio n* of the quartiles of air pollutants and Pollutant Standards Index with upper quartile of tissue factor and thrombomodulin

	Quartile1	Quartile2	Quartile3	Quartile4	P value for linear trend
**PM**_**10**_	50.65-121.74	121.75-178.47	178.48-224.87	224.88-281.55	
**TF**	1:00	1.04(0.8-1.7)	1.10(0.61-1.42)	1.30(1.08-1.40)	0.03
**TM**	1:00	0.91(0.62-2.15)	0.84(0.82-1.7)	0.72(1.07-1.51)	0.04
**CO**	1.54-3.74	3.75-5.74	5.75-8.97	8.98-10.78	
**TF**	1:00	1.05(0.61-1.95)	1.11(0.51-1.72)	1.11(0.91-1.82)	0.06
**TM**	1:00	0.90(0.25-1.81)	0.92(0.51-1.90)	0.91(0.62-1.80)	0.20
**SO**_**2**_	7.54-41.90	41.91-68.24	68.25-77.11	77.12-88.95	
**TF**	1:00	1.04(0.71-2.52)	1.11(0.61-1.90)	1.10(0.91-1.90)	0.08
**TM**	1:00	0.91(0.41-1.60)	0.95(0.50-1.71)	0.92(0.70-1.81)	0.35
**NO**_**2**_	24.71-57.44	57.45-91.84	91.85-106.71	106.72-120.25	
**TF**	1:00	1.03(0.70-1.54)	1.07(0.80-1.64)	1.11(0.80-1.91)	0.08
**TM**	1:00	0.90(0.41-1.60)	0.91(0.51-1.70)	0.95(0.71-1.82)	0.35
**O**_**3**_	12.71-32.14	32.15-41.75	41.76-50.71	50.72-56.14	
**TF**	1:00	1.05(0.71-1.65)	1.1(0.6-1.4)	1.3(1.08-1.4)	0.03
**TM**	1:00	0.91(0.62-2.15)	0.84(0.81-1.75)	0.72(1.07-1.50)	0.04
**PSI**	45.18-72.64	72.65-96.47	96.48-121.19	121.20-135.11	
**TF**	1:00	1.05(0.82-1.91)	1.10(0.64-1.40)	1.41(1.11-1.62)	0.02
**TM**	1:00	0.90(0.61-1.75)	0.84(0.62-1.45)	0.72(1.08-1.64)	0.04

*: Values represent odds ratio (95%CI) adjusted for age, gender, anthropometric measures, dietary and physical activity habits

TF: Tissue Factor(Pg/mL); TM: Thrombomodulin(μmol/L); PM_10_: particular matter _10(_(μg/m^3^); CO: carbon monoxide(ppm); SO_2_: sulfur dioxide(ppb); NO_2_: Nitrogen dioxide(ppb); O_3_: ozone(ppb); PSI; Pollution Standards Index

## Discussion

This study, which to the best of our knowledge is the first of its kind in the pediatric age group, revealed significant association of air pollutants (notably PM_10 _and O_3 _) and PSI with surrogate markers of endothelial dysfunction in early life. Increased levels of PM_10_, O_3 _and PSI increased the OR of elevated TF and reduced TM.

Findings of different studies, mostly of experimental type, have linked air pollution with various predisposing factors of cardiovascular diseases, i.e. the progression of atherosclerosis, endothelial dysfunction, vasoconstriction, high blood pressure, changes in coagulation system, inflammation, oxidative stress, autonomic imbalance and arrhythmias [28].The latest statement of the American Heart Association provides growing body of evidence about a causal relationship between ultrafine PM (PM_2.5_) exposure and cardiovascular morbidity and mortality [29].

Our findings provide confirmatory evidence for the health effects of PM, however similar to our previous study in the pediatric age group [10]; we found associations between larger particulate matter (PM_10_) and factors associated with atherosclerotic cardiovascular disease. This might suggest the higher susceptibility to the threats of air pollutants in the cardiovascular system of children than adults.

Whatever the underlying pathophysiological mechanisms, acute and chronic exposures to air pollutants, notably PM, have been linked to a wide spectrum of cardiovascular disorders characterized by endothelial dysfunction [30]. Episodic exposure to PM _2.5 _induces vascular injury, reflected in part by depletion of circulating endothelial progenitor cell [31]. Adverse vascular effects of diesel exhaust inhalation occur over different running conditions [32], e.g. PM _2.5 _may be an important environmental risk factor for increased arterial blood pressure [33]. A double-blind study on fifteen healthy men exposed to diesel exhaust (PM concentration 300 μg/m^3^) showed a selective and persistent impairment of endothelium-dependent vasodilatation that occurs in the presence of mild systemic inflammation [34]. A study among seniors showed that increases in black carbon and PM_2.5 _were associated with increases in blood pressure, heart rate, endothelin-1, vascular endothelial growth factor and oxidative stress, along with a decrease in brachial artery diameter [35]. The vascular effects of air pollution are also confirmed in young adults [36] and in children [11] as well.

Although the interaction of coagulation, inflammation and endothelial dysfunction is well documented, but different effects of air pollutants have been documented on these systems, and the mechanisms underlying the associations of air pollutants with endothelial function are unclear. The interaction between the inflammation and coagulation systems is reciprocal, with protein C and TF playing a key role in this process [37,38]. We found significant association between PSI and TF, and among air pollutants the highest correlation was documented for PM_10 _and TF. This finding is consistent with various experimental studies that showed exposure to ultrafine PM increase TF expression in atherosclerotic lesions [39-41]. Study on cultured human smooth muscle cells and monocytes documented dose-dependent increases in TF in response to *in vivo *and *in vitro *exposure to ambient PM_2.5 _[42]. Some studies support potential pro-coagulant and thrombotic effects of PM [40], not only its ultrafine particles but also PM_10 _[43].

Findings of a study on 37 workers in a steel production plant revealed that short-term PM exposure is weakly associated with TM, but it did not show any effect on coagulation system [44]. On the other hand, an experimental study suggested that exposure to the soluble organic fraction of PM and diesel exhaust induced oxidative stress and reduced the PAI-1 production of endothelial cells, but it did not affect the TM production [45]. It is suggested that PM activates circulating monocytes directly or indirectly; it might stimulate other cells as pulmonary endothelial cells and might induce pulmonary and/or systemic inflammation [46]. However in an experimental study, ultrafine PM was associated with prothrombotic changes on the endothelial surface but did not trigger an inflammatory reaction and did not induce microvascular tissue injury [47].

A longitudinal study in the US found significant association between black carbon and markers of endothelial function and inflammation, with larger effect in obese individuals [48]. In the current study, similar to our previous experience among children adolescents [10], the association of air pollutants with biochemical markers remained significant even after adjustment for anthropometric measures. This difference might be because of the cross-sectional nature of our studies and the higher susceptibility of the pediatric age group in our study than older age group in the abovementioned study. Moreover, the genetic differences related to oxidative defense and stress response gene expression [41,48] should be considered as well.

Harmful effects of air pollution are mostly attributed to its PM content [29], however exposure to other air pollutants might have various health consequences [49]. Different air pollutants can have independent and possibly synergistic or opposed effects with each other and with PM; and the health impact of exposure to combinations of air pollutants remains to be determined [29].

In an experimental study, asbestos and mineral fibers affected TM level in human umbilical vein endothelial cells [50]. Furthermore, it is documented that a single O_3 _exposure might induce significant biological response in TM level, inflammatory and pro-coagulant reactions in the lungs of mice [18]. Consistent with this study, we found that O_3 _level was significantly associated with TF and TM. Although the only significant relationships of TM with air pollutants were its inverse associations with O_3 _followed by CO, but in higher quartiles of PSI, PM_10 _and O_3_, the OR of elevated TM decreased, and vice versa was documented for TF. This finding may have implications for understanding the systemic effects and possible pro-coagulant state induced by air pollutants; additional studies in this regard seem warranted.

Isfahan is the second most polluted industrial city in Iran, where the number of factories, cars and motorcycles is rapidly increasing [24,25]. Although during the time period of the current study, the urban air had a moderate level of PSI in general, but air pollutants had independent association with surrogate markers of endothelial dysfunction. This association might be because of the vulnerability of children to environmental threats, and/or due to considerably high level of PM_10_, being more than twice as high as standard. Furthermore, this association might be the result of the long-term exposure of the children studied to improper air quality year-round. In addition to the effects of air pollution on coronary artery diseases in the elderly and mortality [51,52] as the main focus of many statements, the impact of air pollutants on children's health should be also considered as a public health priority.

### Study limitations& strengths

The findings of the current study should be considered with its limitations. As with all ecological studies, this study is limited by the lack of precise exposure estimates, and that the ambient pollution concentrations may not adequately reflect exposures of individual subjects. Because of the cross-sectional nature of the study, cause-effect relations cannot be inferred. We compared our findings in areas with different levels of air pollution, repeated measures design particularly capturing episodes of mild and severe pollution might provide more information. Noteworthy to mention that the existing equipment was unable to measure more specific particles such as PM_2.5_, although we found significant association of larger particle (PM_10_) with biomarkers studied, but studying ultrafine particle might result to stronger associations. Moreover, we measured systemic biomarkers, more localized investigation e.g. assessment of lung tissue inflammatory response in broncho-alveolar lavage may yield better results.

The strengths of this study are mainly its novelty in the pediatric age group and in assessment of potential confounding factors and controlling them in our analysis for studying the independent association of surrogate markers of endothelial dysfunction with air pollutants in a representative population-based sample of healthy children.

## Conclusion

The independent association of air pollutants with surrogate markers of endothelial dysfunction and a possible pro-coagulant state is underscored. The presence of these associations with PM_10_, larger than PM_2.5 _usually considered as harmful, and in a moderate air quality, which is commonly considered with few or no health effect for the general population, highlights the need to re-examine environmental health policies and standards for the pediatric age group. Further studies on the effects of air pollution on the first stages of atherosclerosis in early life are needed. Concerns about the harmful effects of air pollution on children's health should be considered a top priority for public health policy; it should be underscored in primordial and primary prevention of chronic diseases.

## Abbreviations

PSI: Pollutant Standards Index; PM: Particular matter; TF: Tissue factor;TM: Thrombomodulin; SO_2_: Sulfur dioxide; O_3: _Ozone; NO_2_: Nitrogen dioxide; CO: Carbon monoxide; ELISA: Enzyme-linked immunosorbent assay; SD: Standard deviation; OR: Odds ratio

## Competing interests

The authors declare that they have no competing interests.

## Authors' contributions

PP participated in the design and conducting the study and helped to draft the manuscript. RK participated in the design and conducting the study and helped to draft and edit the manuscript. AL helped in conducting the study. MM participated in the design of the study and its conduction. SHJ participated in the design of the study and its conduction. RA participated in the design and conducting the study. MMA participated in the design of the study and helped to revise the manuscript. FM participated in the design of the study and helped to revise the manuscript. AA helped in conducting the study. BS participated in conducting the study. All authors read and approved the final manuscript.

## Pre-publication history

The pre-publication history for this paper can be accessed here:

http://www.biomedcentral.com/1471-2458/11/115/prepub
